# SQ-standardized house dust mite immunotherapy as an immunomodulatory treatment in patients with asthma

**DOI:** 10.1111/j.1398-9995.2010.02451.x

**Published:** 2011-02

**Authors:** G Blumberga, L Groes, R Dahl

**Affiliations:** 1 Aarhus University HospitalAarhus; 2 ALKHørsholm, Denmark

**Keywords:** allergic asthma, bronchial hyperresponsiveness, house dust mite, immune response, immunotherapy

## Abstract

**Background:**

Specific immunotherapy is the only treatment with the potential to prevent progression of the allergic disease and the potential to cure patients. The immunomodulatory ability of SQ-standardized house dust mite (HDM) subcutaneous immunotherapy (SCIT) was investigated in patients with allergic asthma.

**Methods:**

Fifty-four adults with HDM-allergic asthma were randomized 1 : 1 to receive SQ-standardized HDM SCIT (ALK) or placebo for 3 years. At baseline, and after 1, 2 and 3 years of treatment, the lowest possible inhaled corticosteroid dose required to maintain asthma control was determined, followed by determinations of nonspecific and HDM-allergen-specific bronchial hyperresponsiveness, late asthmatic reaction (LAR), immediate and late-phase skin reactions, and immunological response.

**Results:**

SQ-standardized HDM SCIT provided a statistically significantly higher HDM-allergen tolerance (*P* < 0.05 *vs* placebo) in terms of a 1.6-fold increase in PD_20_ (HDM-allergen inhalation challenge), a 60-fold increase in skin test histamine equivalent HDM-allergen concentrations, reduced immediate- and reduced or abolished late-phase skin reactions, as well as fewer patients with LAR. PD_20_ (methacholine inhalation challenge) increased initially and was similar between groups. House dust mite SCIT induced an initial increase in serum HDM-allergen-specific IgE (*P* = 0.028 *vs* placebo), which then declined to baseline value. House dust mite SCIT induced an increase in components blocking IgE binding to allergen [ΔIgE-blocking factor: 0.31; 95% CI of (0.26; 0.37)] after 1 year that remained constant after 2 and 3 years (*P* < 0.0001 *vs* placebo).

**Conclusion:**

SQ-standardized HDM SCIT induced a consistent immunomodulatory effect in adults with HDM-allergic asthma; the humoral immune response was changed and the HDM-allergen tolerance in lung and skin increased.

Asthma prevalence is increasing with an estimate of 300 million individuals affected worldwide (1). Asthma is a chronic inflammatory disorder of the airways associated with airway hyperresponsiveness. This causes recurrent episodes of wheezing, breathlessness, chest tightness and coughing usually associated with airflow obstruction in the lungs (1). Asthma progression often involves airway remodelling (permanent structural changes in airways), which may cause irreversible airflow obstruction and is associated with poorer clinical outcome among patients with asthma (2). Airborne allergens such as house dust mite (HDM) allergens are strongly associated with asthma. House dust mite sensitization commonly initiates the allergic disease manifested as allergic rhinitis that can progress to allergic asthma. Because of the allergic march, only few patients with allergic asthma are still monosensitized as adults (3, 4).

Asthma control is usually achieved by treatment with inhaled corticosteroids (ICS). Prolonged use of high doses of ICS may be associated with a risk of systemic side effects, and although treatment with ICS improves symptoms and inhibits exacerbations, this treatment is not curative (1). Therefore, a treatment is needed that can reduce the use of high doses of ICS, prevent asthma progression to a more severe state and potentially cure patients.

Treatment with specific immunotherapy (SIT) induces immune tolerance to the allergen to which the patient is allergic and is currently the only treatment with the potential to alter the natural course of the disease (5, 6). Specific immunotherapy has been shown to be effective in patients with allergic asthma in terms of reducing asthma symptom score and medication requirements, and improve bronchial hyperresponsiveness (BHR) (7). Specific immunotherapy has also been shown to prevent progression of allergic rhinitis into asthma (8, 9) and to provide sustained effect after treatment cessation in allergic patients (8, 10, 11). As concluded in the recent Cochrane collaboration report, trials are required to elicit the effect of SIT compared with other therapies, as well as the effect of SIT with concurrent steroid therapy in patients with allergic asthma (7).

Previously, we reported that 3 years of SQ-standardized HDM subcutaneous immunotherapy (SCIT) was generally well tolerated and partly replaced the need for ICS treatment to control asthma in adults with HDM-allergic asthma; HDM SCIT had a steroid-sparing effect (12). From the same trial, we now present the difference in treatment efficacy between patients treated with SQ-standardized HDM SCIT plus ICS and patients treated with placebo plus ICS in terms of nonspecific and HDM-allergen-specific BHR, late asthmatic reaction (LAR), lung function, HDM-allergen-specific immediate- and late-phase skin reactions and immunological response (HDM-allergen-specific IgE and IgE-blocking factor).

## Materials and methods

### Patients

A total of 54 out of 112 screened patients aged 18–60 were included: 32 men and 22 women. Details of inclusion and exclusion criteria are described elsewhere (12). In short, eligible patients had:

- HDM allergy and perennial asthma (medical history consistent with HDM allergy).
- Regular asthma symptoms requiring long-term treatment with inhaled ICS of at least daily doses of 500–2000 μg fluticasone propionate to control asthma.
- A forced expiratory volume in 1 s (FEV_1_) >70% of predicted value.

### Trial design and treatment

Written informed consent was obtained before entering the trial, and the trial was performed in accordance with current GCP standards and the Declaration of Helsinki (13).

Trial design and treatment regimen are described in detail elsewhere (12). In short, it was a 3-year, double-blind, placebo-controlled trial. Patients were randomized 1 : 1 to receive SCIT with Alutard® SQ *Dermatophagoides pteronyssinus* (*D. pteronyssinus*; ALK, Hørsholm, Denmark) or placebo.

Treatment included an ‘8-week’ up-dosing (up to 100 000 SQ-U) and a ‘3-year’ maintenance with injection intervals of 6 ± 2 weeks. The placebo group received histamine injections according to the same dose increase and maintenance schedule (0.00001, 0.0001, 0.001 and 0.01 mg histamine/ml).

Patients were also treated with ICS to control their asthma, giving two treatment groups: HDM SCIT plus ICS and placebo SCIT plus ICS. The lowest possible dose of ICS that maintained asthma control in each patient was determined by a stepwise reduction protocol at baseline and after 1, 2 and 3 years of treatment (September–December). Asthma control was defined as ‘the ICS dose one step higher than when patients had uncontrolled symptomatic asthma’. Rescue medication (Salbutamol) was allowed as needed. Tolerability was evaluated by adverse event reporting.

The efficacy measures described in the following paragraph were performed at baseline and after 1, 2 and 3 years of treatment, just after the ICS dose adjustment.

### Bronchial challenge tests

*Determination of nonspecific BHR.* The methacholine bronchial challenge test was carried out with the dosimeter method (14) using a Spira Elektro II™ nebuliser (Respiratory Care Centre, Hameenlinna, Finland). Patients inhaled a fixed amount of solution (nebulization time 0.5 s, start of nebulization at 50 ml, working pressure 2 atm), inspiratory flow of 50 l/s; inspiratory volume of 500–800 ml. FEV_1_ was measured before provocation and 90 s after each inhalation. Methacholine was administered in doubling doses from 18 to 11 520 μg. Bronchial challenge was terminated when FEV_1_ decreased at least 20% compared with the patient’s FEV_1_ measured after a saline inhalation. The decrease in FEV_1_ was plotted against the methacholine dose (log scale) and the cumulative dose causing a 20% decrease in FEV_1_ (PD_20_) was determined. The severity of nonspecific BHR was defined as follows: no BHR, PD_20_ > 2000 μg; mild BHR, 1000 μg < PD_20_ ≤ 2000 μg; moderate BHR, 250 μg ≤ PD_20_ ≤ 1000 μg; severe BHR, PD_20_ < 250 μg.

*Determination of HDM-allergen-specific BHR.* The HDM-allergen bronchial challenge test was also carried out with the dosimeter method (14). A dry extract of *D. pteronyssinus* (Aquagen® SQ, 1 000 000 SQ-U/vial) was diluted to concentrations of 1000; 10 000; and 100 000 SQ-U/ml (ALK). Three breathing steps were used for each allergen concentration (two, four and eight breaths) and the cumulated allergen dose for each breathing step was calculated. Baseline FEV_1_ was determined 15 min after two initial inhalations with diluent. The maximum allergen dose delivered was 2 breaths of 1000 SQ-U + 2 breaths of 10 000 SQ-U + (2 + 4 + 8 breaths) of 100 000 SQ-U = 1 422 000 SQ-U. FEV_1_ was measured before and 15 min after each inhalation. Bronchial challenge was terminated when FEV_1_ decreased at least 20% compared with the patient’s baseline FEV_1_. The decrease in FEV_1_ was plotted against the HDM-allergen dose (log scale), and the provocative dose causing a 20% decrease in FEV_1_ (PD_20_) determined. PD_20_ was considered to be 2 844 000 SQ-U if a patient failed to reach a 20% decline in FEV_1_.

*FEV*_*1*_ was measured according to guideline on a daily calibrated dry wedge spirometer (Vitalograph®, Buckingham, UK) and the percentage of predicted value calculated (15).

### Late asthmatic reaction and peak expiratory flow

Patients were provided with an electronic spirometer (Asthma monitor-1; Erish Jaeger GmbH, Hoechberg, Germany) to record their peak expiratory flow (PEF) at home every hour for 24 h after allergen challenge (except during sleep). Late asthmatic reaction was defined as a surplus administration of beta-2 agonist because of asthma symptoms or a fall in PEF of at least 15% from the maximum value after recovery from the early-phase reaction. Morning and evening PEF was recorded over the entire trial.

### Skin prick test titration

Dilutions of 1, 10 and 100 Histamine Equivalent in Prick (HEP) of *D. pteronyssinus* allergen extract (ALK) were used for skin prick test titration (SPTT) on the volar side of the forearm. Positive and negative controls were diluents with or without histamine dihydrochloride (10 mg/ml). Skin reactions (weal area) were measured after 10 min (controls) and after 15 min (HDM-allergen extracts) (New Genius Scanner 4500; Software Genius Inc, Iselin, NJ, USA) (16).

### Intradermal allergen challenge

Intradermally, 0.02 ml (20 SQ-U) of Aquagen® SQ *D. pteronyssinus* (1000 SQ-U/ml; ALK) was injected in the skin of the forearm and a negative control injected in the opposite arm. Immediate-phase skin reactions were determined after 15 min and late-phase skin reactions were determined after 24 h (New Genius Scanner 4500; Genius) (16). The size of the weal area was classified as: (i) no response: <1 cm^2^; (ii) moderate response: 1–20 cm^2^; (iii) severe response: >20 cm^2^.

### Immunological response

Serum specific *IgE* against *D. pteronyssinus* was determined using Magic Lite® SQ (ALK).

Serum specific *IgE-blocking factor* against *D. pteronyssinus* was determined using the ADVIA Centaur immunoassay system (Siemens Medical Solutions, Diagnostics, Tarrytown, NY, USA): IgE-blocking factor was calculated as: 1 – (competed specific IgE/specific IgE); specific IgE is the ordinary determination: the total amount of allergen-specific IgE antibodies that bind to allergen without competing antibodies/components in the solution; competed specific IgE is the total amount of allergen-specific IgE antibodies that bind to allergen in the potential presence of competing components; IgE-blocking factor is the inhibiting capacity of competing components to specific IgE-allergen binding and varies theoretically from 0 (no presence of IgE-blocking components) to 1 (all allergen-specific IgE antibodies are blocked from binding to allergen) (17).

### Statistical analysis

Statistical analyses were performed on the full analysis set (FAS) using the available data without imputation of missing values. Endpoints were tested on a 5% significance level, and all tests and confidence intervals (CI) were two-sided. The null hypothesis was no difference between the two groups. There was no adjustment for multiplicity. Doses of methacholine (μg) and HDM-allergen (SQ-U), as well as the concentration of specific IgE (kU/L) were log_10_-transformed for normality. These data and serum IgE-blocking factor were analysed in anova with change from baseline as response variable, treatment as fixed effect and baseline as covariate. The proportions of patients experiencing a LAR were tested using Fischer’s exact test with exact 95% confidence intervals. In the SPTT, the HDM-allergen dose (in HEP) and skin reactions (weal area in cm^2^) were log_10_-transformed for normality. A statistical parallel line regression model was applied to obtain the histamine equivalent HDM-allergen concentration. The immediate- and late-phase skin reactions were analysed with nonparametric Wilcoxon Rank Sum Test. sas® statistical software, Version 8.2 (SAS Institute, Cary, NC, USA) was used.

## Results

### Patients and tolerability

We included 54 patients with HDM-allergic asthma; 26 patients received HDM SCIT plus ICS (SCIT group) and 28 patients received placebo SCIT plus ICS (placebo group). These two groups were comparable with regard to baseline characteristics (Table 1). Treatment was generally well tolerated; no life-threatening or other serious adverse events related to treatment were reported. Three patients withdrew because of adverse events (two intercurrent illnesses and one worsening of condition) and three patients withdrew because of pregnancies. The trial was completed by 20 patients in the SCIT group and 22 patients in the placebo group. Further details on patient characteristics, safety results and the trial flow according to the CONSORT statement (18) are described elsewhere (12).

**Table 1:** Baseline characteristics (FAS)

	HDM SCIT *N* = 26	Placebo *N* = 28
Sex		
Female	15	17
Males	11	11
Age (years, mean ± SD)	29.8 ± 10.7	28.5 ± 7.1
Asthma severity		
Moderate persistent (step 2.2 and 2.3)*	20	22
Severe persistent (step 2.4)†	6	6
Asthma duration (years, mean ± SD)	14.8 ± 9.7	14.1 ± 6.9
Morning PEF‡ (mean ± SD)	511 ± 111	499 ± 80.1
ICS dose (μg/day)		
500	8	13
750	1	6
1000	11	3
1500	4	5
2000	2	1

FAS, full analysis set; HDM, house dust mite; ICS, inhaled corticosteroid; PEF, peak expiratory flow; SCIT, subcutaneous immunotherapy. All patients were caucasians.

* According to GINA, asthma severity is moderate when the ICS dose required for asthma control is >500 μg and ≤1000 μg fluticasone propionate/day.

† According to GINA, asthma severity is severe when the ICS dose required for asthma control is >1000 μg fluticasone propionate/day.

‡ Baseline PEF was measured during 4 weeks in January. FEV_1_ was <70% of predicted in all patients.

### Bronchial hyperresponsiveness to inhaled methacholine and lung function

**Figure 1:**
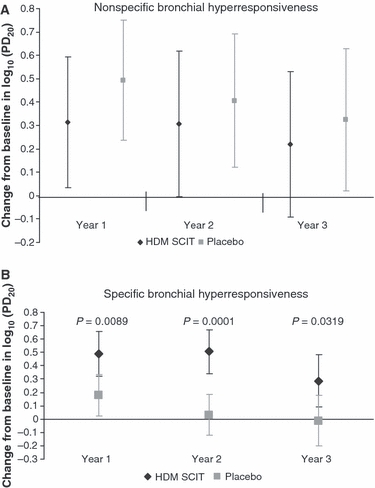
(A) The nonspecific bronchial hyperresponsiveness (BHR) in terms of change from baseline in log_10_(PD_20_); estimate and 95% confidence intervals with *P*-values for the difference between treatment groups. PD_20_ is the methacholine dose (μg) causing a 20% decline in FEV_1_. Test: anova with change from baseline as response variable, treatment as fixed effect and baseline as covariate. (B) The house dust mite (HDM)-allergen-specific BHR in terms of change from baseline in log_10_(PD_20_); estimate and 95% confidence intervals with *P*-values for the difference between treatment groups. PD_20_ is the HDM-allergen dose (SQ-U) causing a 20% decline in FEV_1_. Test: anova with change from baseline as response variable, treatment as fixed effect and baseline as covariate.

At baseline, 42% in the SCIT and 64% in the placebo group experienced moderate to severe BHR to inhaled methacholine (PD_20_ ≤ 1000 μg methacholine) despite receiving high doses of ICS (500–2000 μg) to control their asthma. PD_20_ increased from baseline to 1 year in both the SCIT and the placebo groups and stayed constant after 2 and 3 years. The change from baseline in log_10_(PD_20_) was similar between treatment groups after 1, 2 and 3 years (Fig. 1A).

Morning and evening PEF was unchanged in both treatment groups after 1, 2 and 3 years (data not shown).

### Bronchial hyperresponsiveness to inhaled HDM-allergen

At baseline, 44 patients (81%) had at least a 20% decline in FEV_1_ after HDM-allergen challenge despite receiving high doses of ICS (500–2000 μg) to control their asthma.

In the SCIT group, PD_20_ increased from baseline to 1 year [Δlog_10_(PD_20_): 0.49; 95% CI of (0.32; 0.66)], stayed constant after 2 years and the then slightly declined (Fig. 1B). This corresponded to an initial increase by a factor 1.6 from a median PD_20_ of 552 SQ-U to 857 SQ-U. PD_20_ slightly increased in the placebo group from baseline to 1 year [Δlog_10_(PD_20_): 0.18; 95% CI of (0.020; 0.33)] and then declined to baseline value (Fig. 1B).

The differences in change from baseline between groups were statistically significant, in favour of SCIT, for all 3 years (*P* = 0.0089; *P* = 0.0001; *P* = 0.0319) (Fig. 1B).

### Late asthmatic reaction

**Figure 2:**
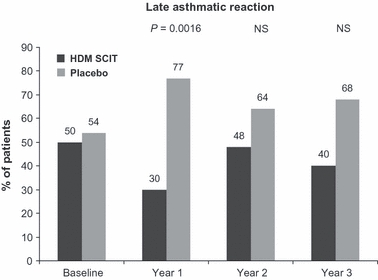
The percentage (%) of patients experiencing a late asthmatic reaction in terms of use of beta-2 agonist in each treatment group with *P*-values for the difference between treatment groups (Fischer’s exact test). NS, no statistical significance.

**Figure 3:**
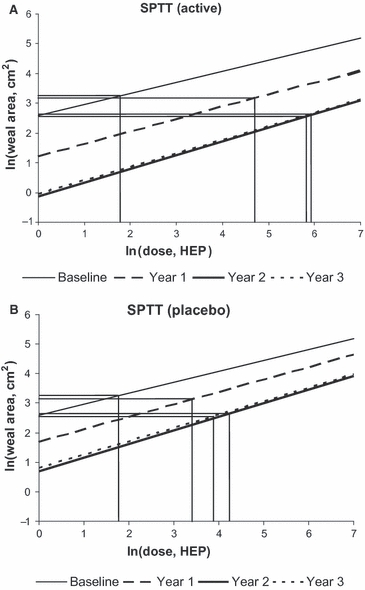
In the skin prick test titration, the house dust mite-allergen dose (in HEP) and skin reactions (weal area in cm^2^) were ln-transformed. A statistical parallel line regression model was applied. The histamine equivalent *Dermatophagoides pteronyssinus* allergen concentrations at baseline and after 1, 2, and 3 years of active treatment (A) and placebo treatment (B) are plotted. HEP, Histamine Equivalent in Prick.

At baseline, 28 patients (52%) experienced a LAR; 13 of these patients received SCIT and 15 received placebo. After 1 year of treatment, seven patients in the SCIT group [30%; 95% CI of (13; 53)] and 20 patients in the placebo group [77%; 95% CI of (56; 91)] experienced a LAR (Fig. 2; *P* = 0.0016 *vs* placebo). The percentage of patients with a LAR in the SCIT group was also lower than in the placebo group after 2 and 3 years (Fig. 3).

### Skin prick test titration

At baseline, the estimated HDM-allergen concentration that caused histamine equivalent skin reactions was similar between groups (Fig. 3). This concentration increased from 6 to 377 HEP in the SCIT group and from 6 to 48 HEP in the placebo group after 3 years of treatment (Fig. 3); the difference between groups was statistically significant for all 3 years (*P* < 0.0001).

### Intradermal allergen challenge

**Figure 4:**
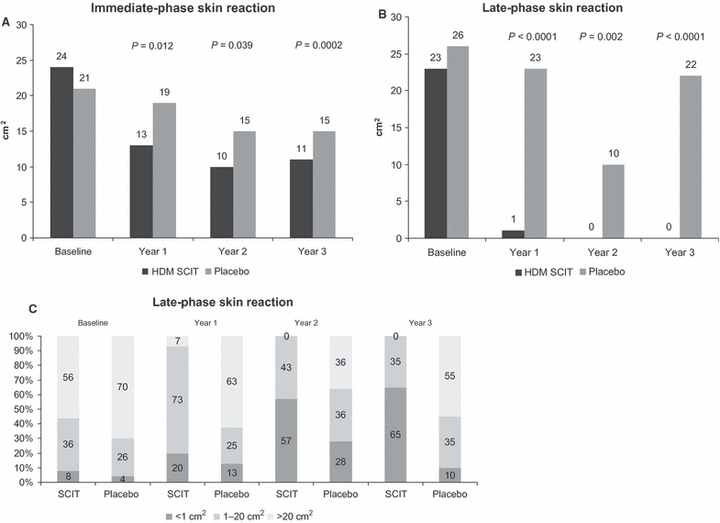
(A) Median areas (in cm^2^) of the immediate-phase skin reactions for each treatment group at baseline and after 1, 2 and 3 years of treatment with *P*-values for the difference between treatment groups (Wilcoxon Rank Sum Test). (B) Median areas (in cm^2^) of the late-phase skin reactions for each treatment group at baseline and after 1, 2 and 3 years of treatment with *P*-values for the difference between treatment groups (Wilcoxon Rank Sum Test). (C) The percentage (%) of patients without a late-phase skin reaction (weal area <1 cm^2^), the percentage of patients that experienced intermediate reactions (weal area 1–20 cm^2^) and the percentage of patients that experienced a severe reaction (weal area >20 cm^2^) are illustrated at baseline, and after 1, 2 and 3 years of treatment for both house dust mite (HDM) subcutaneous immunotherapy (SCIT) and placebo treatment groups.

At baseline, the immediate-phase skin reactions were similar between the two groups: a median weal area of 24 cm^2^ in SCIT *vs* 21 cm^2^ in placebo. After 1 year of SCIT, the reaction was reduced to a median weal area of 13 cm^2^, after 2 years to 10 cm^2^, which remained constant after 3 years of SCIT (11 cm^2^). This reduction was statistically significantly higher than in the placebo group after all 3 years of treatment (*P* < 0.04) (Fig. 4A).

At baseline, the late-phase skin reactions were similar between the two treatment groups: a median weal area of 23 cm^2^ in SCIT *vs* 26 cm^2^ in placebo. In the SCIT group, the late-phase skin reaction was reduced to 1 cm^2^ after 1 year and 0 cm^2^ (no reaction) after 2 and 3 years. As a contrast, this reaction was unchanged in the placebo group and statistically significantly different from that in the SCIT group after all 3 years (*P* < 0.002) (Fig. 4B).

In the SCIT group, the percentage of patients without a late-phase skin reaction increased from 8% at baseline to 65% after 3 years; none of the patients had a severe reaction (weal area >20 cm^2^) after 2 and 3 years (Fig. 4C). In contrast, the percentage of patients with a late-phase skin reaction in the placebo group was constant over 3 years and 55% experienced a severe reaction after 3 years (Fig. 4C).

### Immunological response

In the SCIT group, the change from baseline to 1 year in serum specific IgE (*D. pteronyssinus*) [Δlog_10_(IgE): 0.048; 95% CI of (−0.017; 0.11)] was statistically significantly different from that in the placebo group [*P* = 0.028; Δlog_10_(IgE): −0.051; 95% CI of (−0.11; 0.0080)]. In the SCIT group, specific IgE declined to baseline value after 2 and 3 years, and the difference between treatment groups at year 2 and 3 was statistically insignificant.

**Figure 5:**
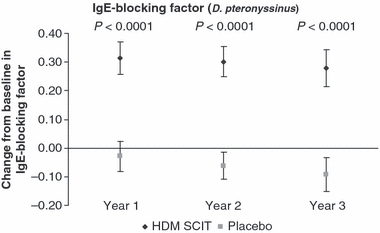
Change from baseline in IgE-blocking factor (*Dermatophagoides pteronyssinus*); estimate and 95% confidence intervals after 1, 2 and 3 years of treatment with *P*-values for the difference between treatment groups. IgE-blocking factor is a measure of the inhibitory capacity of competing components to specific IgE-allergen binding. Test: anova with change from baseline as response variable, treatment as fixed effect and baseline as covariate.

In the SCIT group, the increase from baseline to 1 year in serum specific IgE-blocking factor (*D. pteronyssinus*) [ΔIgE-blocking factor: 0.31; (0.26; 0.37)] remained constant after 2 and 3 years (Fig. 5). There was no change from baseline in the placebo group and a statistically significant difference between treatment groups, in favour of SCIT, was found for all 3 years (*P* < 0.0001) (Fig. 5).

## Discussion

Treatment with SIT induces immune tolerance to the allergen to which the patient is allergic and is the only treatment with the potential to alter the natural course of the disease (5, 6). Patients with HDM-allergic asthma were treated with HDM SCIT plus ICS or placebo plus ICS for 3 years. Each year, ICS was adjusted to the minimal dose that maintained the patient’s asthma in control and the efficacy and tolerability of SCIT was evaluated accordingly. We previously reported that SCIT reduced the ICS dose that maintained asthma in control and was well tolerated in HDM-allergic patients with moderate asthma (12).

We found that nonspecific BHR was reduced similarly in both groups after 1 year. Methacholine acts as direct stimuli of BHR by binding to specific receptors on the bronchial smooth muscle to cause constriction (19). Regular treatment with ICS is known to progressively reduce the patient’s sensitivity to this stimuli (20), and we found that SCIT fully replaced this effect over 3 years. However, nonspecific BHR was not fully reversed in any of the groups; persistent BHR has been found to correlate significantly with airway remodelling (21), which may be irreversible once established (22). Lung function was also unchanged in both groups after 3 years of treatment. Overall, supporting previous findings that HDM SCIT partly replaced ICS in providing asthma control (12).

Both immediate- and late-phase reactions to HDM allergens were markedly more reduced in SCIT plus ICS treated patients than in patients treated only with ICS. House dust mite-allergens act indirectly by inducing the allergic reaction causing BHR and skin reactions; an immediate IgE-mediated mast-cell-driven response within 15–30 min, and for some, a late-phase allergic inflammation 6–12 h after allergen exposure (19, 23). Measuring allergen-specific BHR and skin reactions to HDM-allergen is, therefore, a direct measure of allergen tolerance in lungs and skin that can indicate if treatment improves the underlying allergic disease manifested as asthma. In this trial, patients treated with SCIT tolerated a higher inhaled HDM-allergen dose when challenged (PD_20_ increased), fewer patients experienced a LAR to inhaled HDM-allergen challenge, the histamine equivalent concentration was higher at SPTT and the immediate-phase skin reaction was reduced. The late-phase skin reactions were strikingly diminished or abolished in the SCIT group and none of these patients experienced a severe late-phase skin reaction after only 2 years of treatment. Conclusively, HDM SCIT provided improved tolerability to HDM allergens in skin and lungs.

After SCIT, the suppression of early reactions in skin has been found associated with reduction in mast-cell numbers (23–25), and suppression of late-phase skin reaction has been found associated with reduction in the number of infiltrating T cells, eosinophils, basophils and neutrophils and inflammatory mediators (23, 26). Thus, the increased allergen tolerability observed in this trial is probably because of a reduced allergic inflammation as a consequence to SCIT improving the underlying allergic disease.

Allergen-specific IgE is considered a central player in the allergic reaction (27) and is increased in serum of patients with allergy (28). In this trial, SCIT also provided a change in the humoral immune response, which was absent in patients treated only with ICS; an initial increase in serum HDM-allergen-specific IgE antibodies followed by a decline and a marked increase in the effect of components blocking HDM-allergen-specific IgE function. The precise mechanism by which SIT acts remains unclear; however, consistent with the findings in this trial, an effect on IL-10 and TGF-β secreting regulatory T cells (Treg) associated with switching of allergen-specific B-cells towards IgG_4_ production and suppression of allergen-specific IgE production is the most probable mechanism (23, 25, 29). This was also observed by others (30, 31), and probably reduce the immediate IgE-mediated mast-cell-driven allergic response and thereby also reduce the release of inflammatory mediators that induce the late-phase response, in this trial observed as reduced immediate- and late-phase reactions in lung and skin.

Treg may also directly inhibit the activation of allergen-specific Th2 cells, thereby reducing the production of Th2-cytokines and their multiple effects on cells involved in the allergic response (23). Chen et al. demonstrated that 1 year of SQ-standardized HDM SCIT significantly decreased the serum level of the Th2-cytokine IL-13 (involved in the pathogenesis of airway remodelling) more than did ICS in children with asthma (32). At the same time, the serum level of IL-4 (a Th2-cytokine) decreased and the serum level of IFN-γ (a Th1-cytokine) increased (32). This shift from an allergic ‘Th2 cell predominance’ to a more normal ‘type 1 Treg cell predominance’ immune response to allergen that also involves a shift in the balance of Th2 and Th1 cytokine expression (6, 23, 25, 29) is also observed in allergic rhinitis after SIT, consistent with the idea of a one-airway-one-disease theory (33–35). Therefore, the prevention of disease progression (8, 9) and sustained effect (8, 10, 11) observed in patients with allergic rhinitis after SIT may also be expected treatment outcomes in patients with allergic asthma.

Because of the added benefit over ICS, we recommend that SIT is considered as first-hand medication when treating patients with HDM-allergic asthma. Because of current knowledge and current guidelines on asthma treatment (GINA) (1), initial treatment is suggested to consist of SIT combined with controller medication in terms of inhaled ICS and reliever medication as needed. This trial indicates that over time, SIT will improve the underlying allergic disease in the majority of patients with asthma; the ICS dose can be down-titrated accordingly. For some patients with asthma, SCIT may ultimately become sufficient to control their asthma in combination with reliever medication.

In conclusion, SQ-standardized HDM SCIT induced a consistent immunomodulatory effect in adults with HDM-allergic asthma in terms of a change in the humoral immune response and as increased HDM-allergen tolerance in the lungs and skin of these patients. This strongly indicates that HDM SCIT treats the underlying allergic disease; sustained effect and prevention of disease progression may be expected treatment outcomes for the patient with HDM-allergic asthma.
